# Development of an RP-UHPLC-PDA method for quantification of free gossypol in cottonseed cake and fungal-treated cottonseed cake

**DOI:** 10.1371/journal.pone.0196164

**Published:** 2018-05-23

**Authors:** Aparecido Almeida Conceição, Clemente Batista Soares Neto, José Antônio de Aquino Ribeiro, Felix Gonçalves de Siqueira, Robert Neil Gerard Miller, Simone Mendonça

**Affiliations:** 1 Instituto Multidisciplinar em Saúde, Universidade Federal da Bahia, Vitória da Conquista, Bahia, Brazil; 2 Embrapa Agroenergia, Brasília, Federal District, Brazil; 3 Universidade de Brasília, Brasília, Federal District, Brazil; Alexandria University, EGYPT

## Abstract

Cottonseed cake biomass, which is a residue of oil extraction, is potentially appropriate for use as animal feed, given the high mineral, fibre and protein content. The presence of free gossypol, however, a toxic pigment in the glands of the cotton plant, limits use of this biomass for monogastric livestock. A promising method to detoxify cottonseed cake relies on fermentation by fungi, which can eliminate up to 100% of gossypol. In order to quantify trace levels of free gossypol in different cotton materials, including cottonseed cake treated with macrofungi, a simple and rapid chromatographic detection method was developed and validated. Under optimized conditions, extraction was performed using 70% acetone. The extract was then analysed by Ultra High-Performance Liquid Chromatography (UHPLC), with gradient elution on a C18 reverse phase column KINETEX^®^ (100 x 2.10 mm, 2.6 μm). Methanol-0.1% TFA aqueous solution was employed as mobile phase and PDA detection conducted at 254 nm. The optimized method was validated by analysis of specificity, linearity and range, limit of detection, limit of quantification, precision and accuracy. Detection and quantification limits were observed at 0.2 and 0.5 μg/mL, respectively. With good reproducibility, with precision (RSD)<10% and recovery greater than 94%, the developed assay was appropriate for quantification of low quantities of free gossypol. The validated method was successfully applied to determine trace levels of free gossypol cottonseed treated with a macrofungus.

## Introduction

Gossypol is a polyphenolic compound that provides protection in cotton (*Gossypium* sp.) against predators that include insects, fungi and nematodes [1–4]. This yellow pigment is produced and stored in specialized glands present in the leaves, seeds, stems and flower buds [5–7]. Biosynthesis of gossypol occurs by peroxidation of hemigossypol, giving rise to two enantiomers: (+)- and (-)-gossypol [8–10]. (±)Gossypol is found in cottonseed cake (CSC) in two forms: as free gossypol (FG) and as gossypol bound to proteins [11]. According to AOCS official methods, FG is defined as all the gossypol content that is extractable with aqueous acetone [12].

The FG content in intact cottonseed (CS) is typically around 0.7% (7000 ppm) [7], whilst the detectable remaining free gossypol in CSC, after cotton oil extraction, varies between 0.1 and 0.6%, when extracted using solvent-based methods. If initial extrusion or expansion steps are employed prior to solvent extraction, however, FG content may appear to be lower, with levels as low as 0.05% documented. Levels can also be sub-estimated when mechanical extraction methods are applied, such as those involving pressure and temperature treatment. In these methods, the detected content of FG in the CSC has also been reported in the region of only 0.05% [7,13].

CSC represents a rich nutrient source, with abundant protein, mineral, fat, and fiber content [14]. However, several animal species, including humans, are sensitive to the toxic effects of FG. Of particular importance are monogastric livestock, which are frequently negatively affected by even low quantities of this polyphenolic pigment [7,15–21]. For example, color alteration has been noted in eggs from chickens fed with feed containing 220 ppm of FG[22], which represents only 0.022% of the feed. The directive 2002/32 of the European Union (2002L00032- February of 2013) states that the maximum FG concentration for cottonseed cake for complete feedstuffs are 20 ppm for laying hens and piglets, 60 ppm for pigs, 100 ppm for poultry and calves, and 500 ppm for cattle, sheep and goats [7]. Given such toxicity and the strict maximum permitted levels for FG, CSC needs to be further processed to decrease the toxic molecule to the permissible levels for non-ruminants feed [23–26].

Chemical, physical and biological treatments have been employed to reduce the toxic effects of FG in CSC [24,27–29]. A promising method for eliminating gossypol is through biodetoxification by filamentous fungi such as *Aspergillus oryzae* [30], *Candida tropicalis*, *Saccharomyces cerevisae*, *Aspergillus niger* [31–33] and *Pleurotus florida* [34]. This process also increases substrate value, with gain in crude protein, total amino acids [31] and antioxidant activities [30], leading to improvement in livestock growth performance [26]. The white rot fungi (Basidiomycetes) have also been shown to be capable of detoxifying up to 100% of pure gossypol when incorporated into growth media [34]. When tested for cottonseed detoxification, such fungi have also been reported to simultaneously decompose pollutants and well as macromolecules such as lignin [35], as well as environmental pollutants such as synthetic and aromatic dyes, polycyclic aromatic hydrocarbons, tri-nitro-toluene (TNT), and polychlorinated biphenyls [36,37].

For precise evaluation of biodetoxification of gossypol, analytical methods that enable accurate quantification of this polyphenol at trace levels are required [38]. In situations demanding quantitation of multiple samples, it is also desirable that methods offer low cost and short sample analysis times. Numerous analytical approaches for detection and quantification of free and total gossypol in different sample matrices have been reported, including spectrophotometric, colorimetric, gravimetric and titrimetric methods [39–52]. Currently, recommended methods for detection and quantification of gossypol are based on liquid chromatography with UV detection [42,53,54]. Such methods, however, are inappropriate for accurate analysis of gossypol in situations where the analyte is present in very low concentrations, such as in fermented cottonseed cake (FCSC). All previous investigations into gossypol detoxification in cottonseed cake have employed only such less sensitive quantitative methods available, which are more appropriate for untreated samples where concentrations of gossypol are considerably higher [26,32,34, 55–57].

Extra attention needs to be applied during FG extraction and detection when dealing with samples containing very low gossypol content. FG can be easily degraded under certain conditions, such as when employing high temperatures [58], extraction with alcoholic solvents or long exposure to oxygen [40]. Such care has not been adopted in many previously published extraction methodologies for FG. In addition to the risk of FG degradation, others factors, such as the employment of complex solvent mixtures [58,59] and time-consuming extraction methods [41,42,60] limits the uptake of a considerable number of published procedures for FG extraction and detection. The official AOCS method indicated to determine free and total gossypol in cottonseed, cottonseed meats and cake is based on High Performance Liquid Chromatography (HPLC). This method, however, employs a long, complex and expensive sample preparation process, which is inappropriate for analysis of large numbers of samples [61].

Considering the sensitivity offered by HPLC, this approach has been employed for accurate analysis of FG content in diverse samples, ranging from glandless cotton plants [42,53,54] to animal and human samples [49,62–64]. The advantages of HPLC also go beyond the high sensitivity, with technical errors minimized due to the low number of steps employed in analysis methods. Recently developed liquid chromatography technologies such as Ultra High-Performance Liquid Chromatography (UHPLC) may enable even further improvement in FG detection, appropriate for accurate analysis of levels in FCSC following treatment for theoretical elimination of gossypol, or for determination of safety for livestock consumption.

In consideration of the need for a highly sensitive, selective, rapid and specific technique for determining trace levels of FG in FCSC, we developed an UHPLC method capable of efficient and rapid extraction and detection of trace level FG in cottonseed cake following detoxification by fungi.

## Materials and methods

### Chemicals

All organic solvents were of HPLC grade. Acetone and methanol were purchased from TEDIA^®^. Trifluoroacetic acid HPLC grade and (±)-Gossypol standard (≥95% purity HPLC) were purchased from SIGMA^®^ (St. Louis, USA). High purity deionized water (resistivity 18.2 MΩ.cm) was obtained by using a Milli-Q Direct system (Millipore, Billerica, MA, USA).

### Cotton material and fungal strain

Processed cottonseed cake was provided by a cotton processing company located in Buritis, MG, Brazil. A white rot basidiomycete fungal strain of a *Pleurotus* sp. was obtained from a local collection of microorganisms and microalgae utilized in agroenergy and biorefinery research applications (Embrapa, Brasília, Federal District, Brazil). This strain, previously identified as an efficient biodetoxifier of FG, was employed to enable validation of the extraction and detection methods developed for detection of gossypol at trace levels following biodetoxification.

### Fungal inoculation on cottonseed cake

Samples of 70 g of CS or CSC with moisture adjusted to 60% were autoclaved in 350 mL Erlenmeyer flasks for 60 min at 121°C. Under aseptic conditions, 5 mm mycelial discs of the *Pleurotus* sp. strain, previously grown on potato dextrose agar with CSC medium for 15 days at 28°C, were inoculated onto both CS and CSC. Inoculated cotton material was then incubated at 28°C for a 30-day period. Colonized material was dried at 65°C for 48 h (dry matter), resulting in fermented cottonseed (FCS) and fermented cottonseed cake (FCSC) samples. All experiments were conducted in triplicate.

### Sample extraction

Extraction was based on the AOCS official method [11], with modifications for optimized sample extraction with regard to solvent, temperature and time of extraction, ultra-sonication and speed vacuum sample concentration. Following autoclaving, or autoclaving followed by fermentation, extraction was conducted for FCS, CSC, autoclaved CSC and FCSC as follows: dried samples were finely ground with an ultra-centrifugal mill and 1 gram samples transferred into 15 mL centrifuge tubes. Extraction was conducted with two sequential cold ultra-sonication steps of 5 minutes, first with 3 mL of ultrapure water, followed by addition of 7 mL acetone directly onto the sample. Tubes were centrifuged at 9000 rpm (8370 rcf) for 5 minutes at 8°C and 2 mL of supernatant then transferred to fresh 15 mL centrifuge tubes. Solvent was evaporated using a speed vacuum at room temperature. The residue was re-suspended with 200 μL of 70% aqueous acetone, transferred into a 1.5 mL microtube and centrifuged at 14000 rpm (20590 rcf) for 10 minutes at 8°C. The supernatant was transferred to a sample vial with glass inserts. Given the high content of FG in CS and autoclaved CS, these samples were not evaporated by speed vacuum (dx.doi.org/10.17504/protocols.io.krncv5e [protocols.io]).

### Ultra High Performance Liquid Chromatography (UHPLC)

A number of different parameters were tested during method development, which comprised: reversed phase columns Acquity^®^ UPLC HSS T3 (2.1 x 150 mm, 1.8 μm), BEH C18 and BEH C8 (2.1 x 150 mm, 1.7 μm), ZORBAX^®^ HPLC SB-C18 and SB-CN (4.6 x 250 mm, 5 μm), column temperature, organic mobile phase (acetonitrile, methanol or mixture of both), elution mode (isocratic or gradient), and modifiers of mobile phase (acetic acid, formic acid, or trifluoracetic acid (TFA)). The final optimized method was performed on a Waters ACQUITY UPLC H-Class system (Waters, Milford, USA) equipped with a Photodiode Array (PDA) detector. Gradient elution was conducted using a mobile phase starting with 40% aqueous (TFA 0.1%, v/v) and 60% organic (100% methanol) solvent on a KINETEX^®^ reversed phase (RP) C18 column (100 x 2.1 mm, 2.6 μm) at 35°C (Table 1). A solvent flow rate of 0.4 mL/min was employed, with a total run time of 14 minutes (including column re-equilibration). The signal was monitored at 254 nm. The sample manager temperature was maintained at 8°C, with a 2 μL sample injected. Data acquisition and integration were performed using Empower (version 3.0, Empower software, Milford, USA) (dx.doi.org/10.17504/protocols.io.krncv5e [protocols.io]).

**Table 1:** Gradient elution applied for free gossypol (FG) detection by UHPLC.

Time (min)	Aqueous (%)	Organic (%)
Initial	40	60
8	0	100
10	0	100
10.01	40	60
14	40	60

### Validation parameters

Validation of the method comprised evaluation of selectivity, linearity, limit of detection, limit of quantitation, precision (repeatability) and accuracy (recovery), in accordance to the International conference of harmonisation guideline Q2(R) [65].

Specificity was assessed by comparing the retention time of the analyte in the cottonseed cake sample to retention time of the gossypol standard. Gossypol identity was also confirmed based on the purity spectra of the peak extracted from a cottonseed cake sample fortified with standard solution (50 μg/mL).

Linearity was evaluated using a calibration curve of gossypol, in the range of 0.5 to 100 μg/mL (0.5, 1, 5, 10, 25, 50, 75, 100 μg/mL). Gossypol standard was firstly solubilized in chloroform (5 mg/mL) and then diluted in 70% aqueous acetone solution to obtain standard concentrations. Curves of peak area versus concentration were constructed by means of a least squares method. Coefficient of determination and residuals were evaluated for fitting data to the model.

Limit of detection (LOD) and limit of quantification (LOQ) were determined by injecting gossypol standard solutions in the range of 0.05 to 1 μg/mL. Chromatograms were visually inspected and signal-to-noise ratios were calculated. The LOD was defined as the lowest concentration with ratio signal-to-noise greater or equal to three. The LOQ was defined as the lowest concentration with ratio signal-to-noise greater or equal to 10, with acceptable precision and accuracy.

For analysis of precision, the repeatability of the analytical method was assessed in six replicate determinations of gossypol in a homogeneous cottonseed cake sample. Inter-day precision was assessed by analyzing the same homogeneous sample over three consecutive days. Relative Standard Deviation (RSD) was calculated for each determination series, using acceptance criteria of RSD≤ 15% for repeatability. Inter-day precision was evaluated by one-way analysis of variance (ANOVA) at the 5% significance level.

Accuracy was determined by a recovery test. Three sets of samples were evaluated: standard solutions at concentrations of 2 μg/mL (low), 20 μg/mL (medium), and 80 μg/mL (high) in 70% aqueous acetone, 1 g of CSC and 1 g of CSC fortified with the standard. The experiment was conducted over three consecutive days. Absolute recovery was calculated after extraction and analysis of all sample sets.

## Results

### Optimized extraction

**Fig 1:**
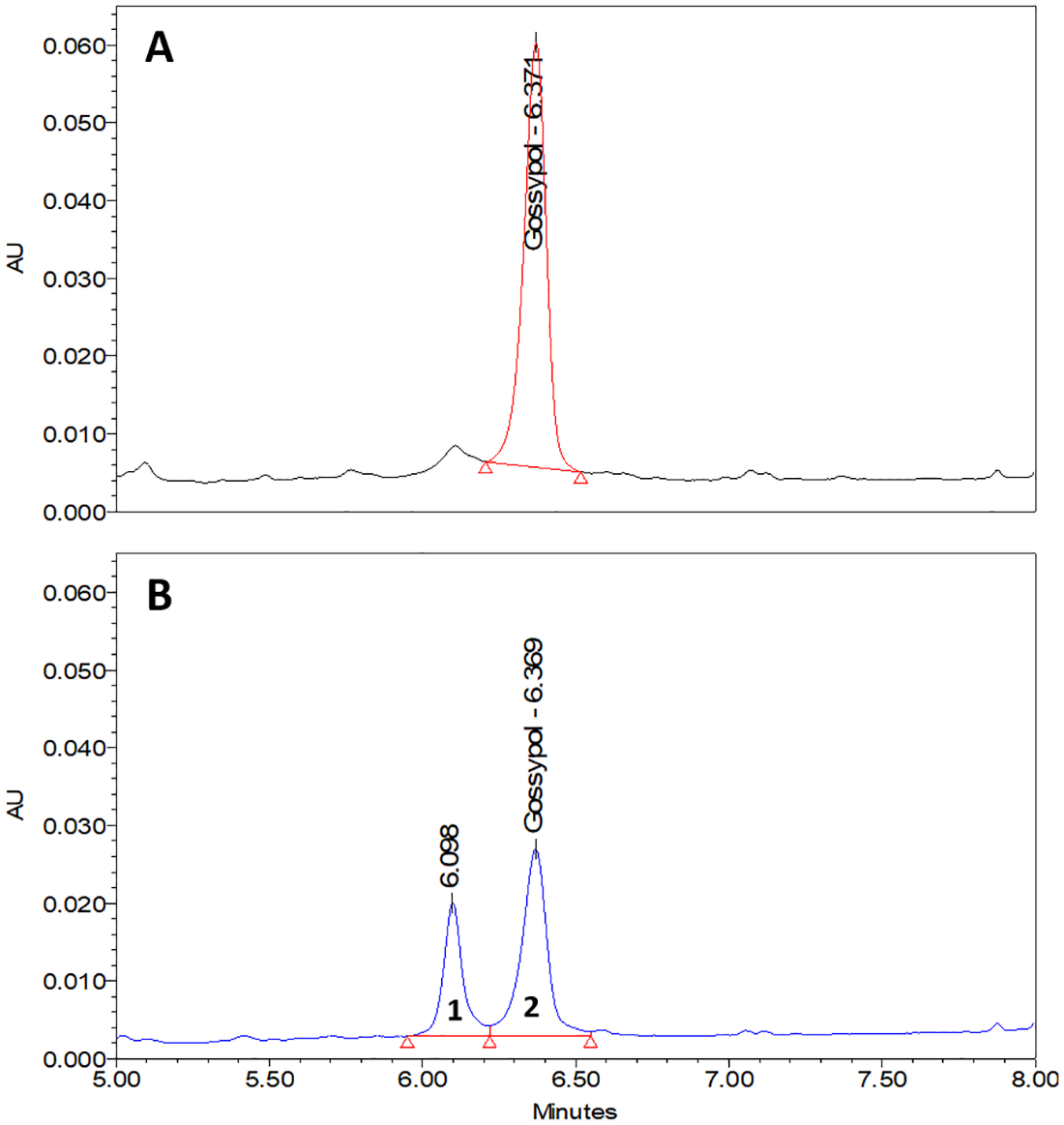
UHPLC chromatograms of free gossypol (FG) extracted from cottonseed cake (CSC) using different acetone concentrations. Identical gradient elution conditions and extraction conditions with sonication on ice were applied throughout. For both samples, 1g CSC were spiked with 80 μL of a 5 mg/mL gossypol solution. (A) Extraction with 70% acetone (peak area = 282558); (B) extraction with 100% acetone (peak 1 area = 142704, peak 2 area = 77803).

**Fig 2:**
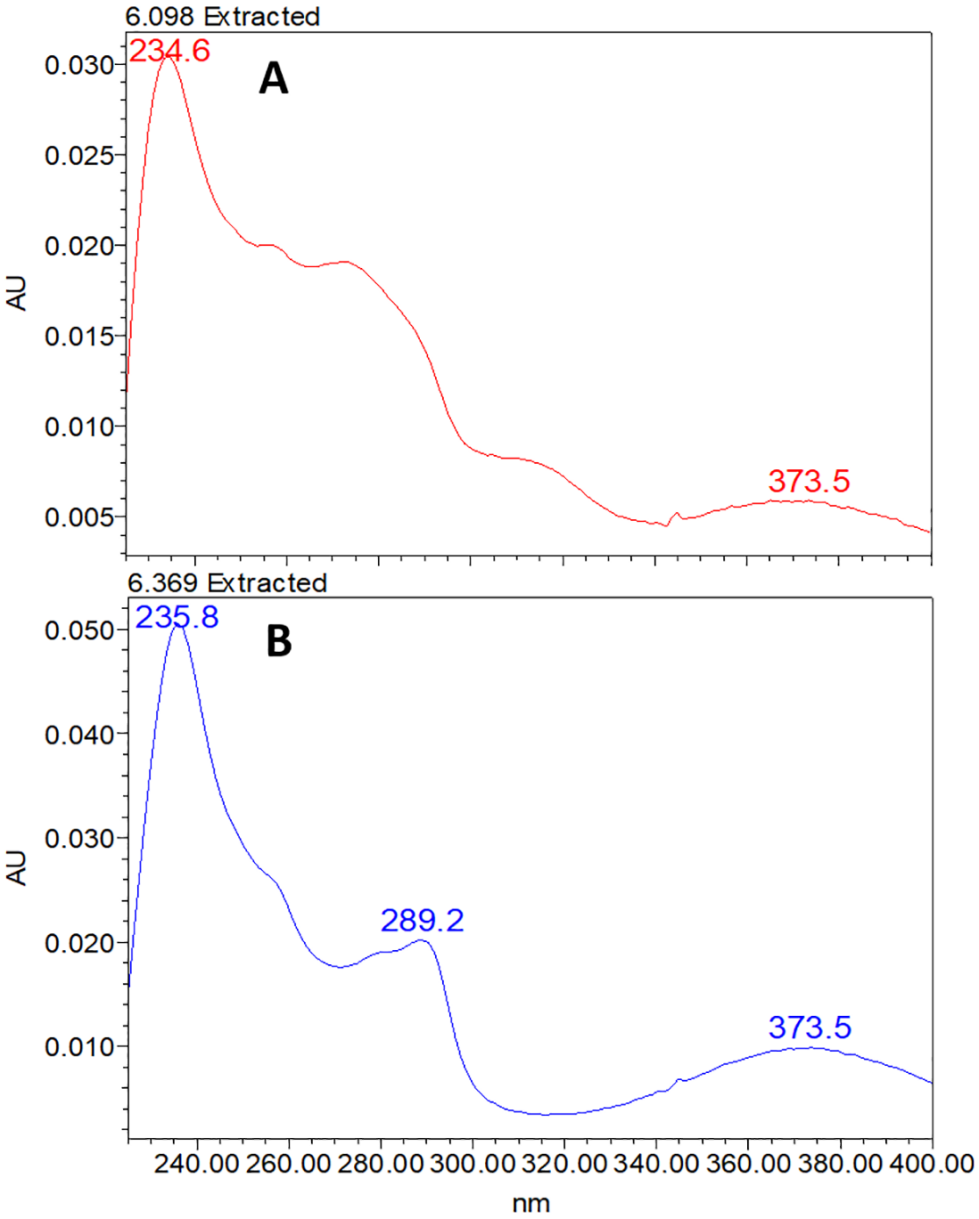
Spectrum extracted from peaks observed in Fig 1B. The spectrum was extract from the respective retention times of the peaks observed in Fig 1B. (A) Spectrum from peak 1 (6.098 min); (B) spectrum from peak 2 (6.369 min).

The principle objective of this study was to optimize extraction of FG in FSCM whilst assuring molecule stability. For this, numerous procedures were evaluated, including sonication with or without ice, evaporation on a rotary evaporator or a speed vacuum, and extraction using different solvents. Extraction with 100% acetone or 70% acetone rendered the greatest amount of FG. However, when extraction was performed using a CSC sample spiked with gossypol standard (40μg/μL), an unknown peak near to that for gossypol (Fig 1B) occurred when using 100% acetone, in contrast to its absence when using 70% acetone (Fig 1A). In Fig 2, the spectrum of the two peaks from Fig 1B are compared, revealing that both substances are correlated, with peak 1 as a result of gossypol degradation (peak 2). Based on these findings, employment of a final concentration of 70% acetone (application of 3 mL of water, followed by 7 mL of acetone) under cold ultra-sonication conditions, was demonstrated to be appropriate for extraction of FG from samples whilst maintaining gossypol stability.

**Fig 3:**
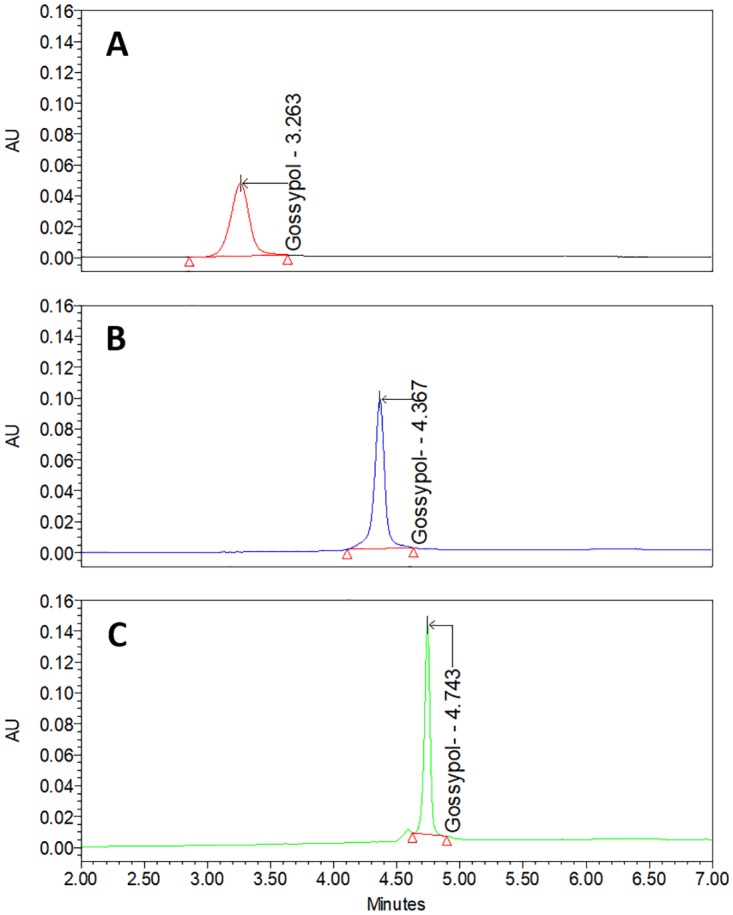
UHPLC chromatograms of standard gossypol generated following comparison of different elution organic phases. (Gradient elutions with organic phases composed of (A) 100% acetonitrile (ACN), (B) 50% ACN/50% methanol (MeOH), and (C) 100% MeOH.

Different columns, temperatures, elution’s gradients and solvents were compared for the optimization of elution for gossypol detection by UHPLC. Acquity^®^ UPLC HSS T3 (2.1 x 150 mm, 1.8 μm), BEH C18 and BEH C8 (2.1 x 150 mm, 1.7 μm), ZORBAX^®^ HPLC SB-C18 and CB-CN (4.6 x 250 mm, 5 μm) columns all resulted in only broad peaks for gossypol, as well as inappropriately long elution time periods. The KINETEX^®^ C18 column presented narrow peaks for gossypol with a short elution time. Column temperature was also a factor that interferes in the gossypol elution process. Temperatures above 40°C resulted in a decrease in the peak resolution when compared to elution at 35°C. The employment of 100% methanol (MeOH) (Fig 3C) as eluting solvent resulted in an optimal peak shape when compared with 100% acetonitrile (ACN) (Fig 3A) and a mixture of 50% MeOH and 50% ACN (Fig 3B).

### Validation

**Fig 4:**
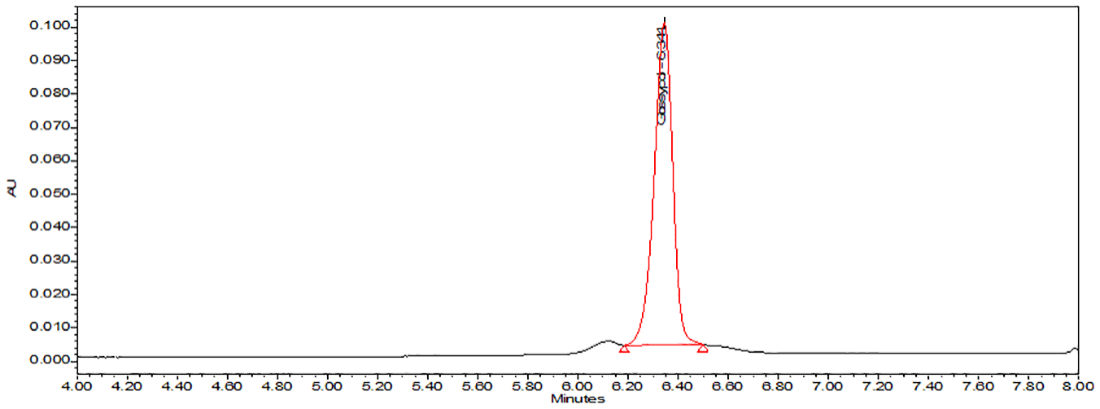
UHPLC chromatogram of cottonseed sample spiked with standard gossypol (50 μg/mL). A retention time of 6.3 min was observed for the gossypol peak. Gossypol was analyzed on a KINETEX^®^ C18 column using a gradient elution with solvent consisting of 0.1% TFA in methanol-water. Absorbance was measured at 254 nm.

An optimal sensitivity and peak shape was obtained when performing gradient elution with methanol and 0.1% aqueous TFA as mobile phase, and a KINETEX^®^ reversed phase C18 column at 35°C. The retention time for the molecule was observed at 6.3 min (Fig 4).

The RP-UPLC-PDA method showed good linearity in the range of 0.5 μg/mL to 100 μg/m with R^2^>0.999. Detection and quantitation limits were 0.2 μg/mL and 0.5 μg/mL, respectively. At low, medium and high levels, recovery was greater than 94%, with good precision (RSD<10%, n = 6).

**Fig 5:**
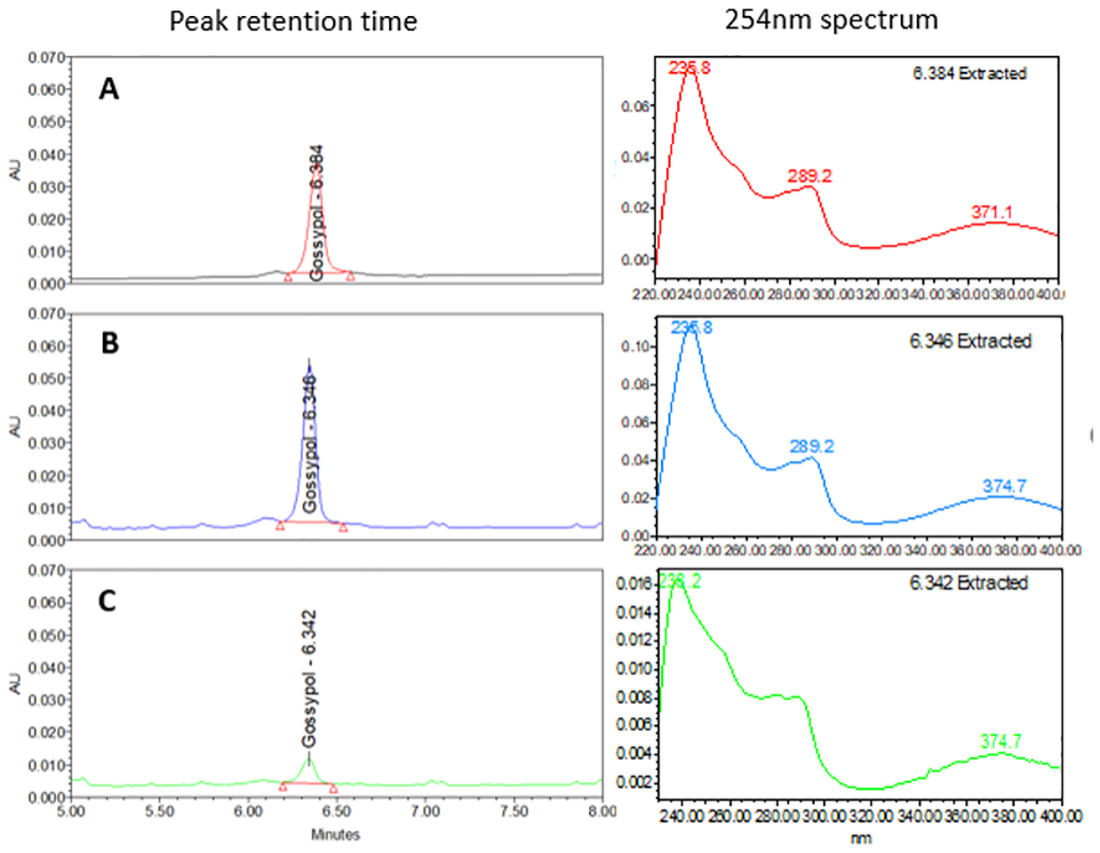
UHPLC chromatograms and respective spectra at 254 nm of gossypol from standard solution and cottonseed sample extracts. (A) Standard gossypol at 40μg/mL and its spectrum extracted at 6.384 min; (B) cottonseed sample spiked with standard gossypol (40μg/mL) and spectrum at 6.346 min; (C) cottonseed sample extract and spectrum at 6.342 min.

Specificity and identity confirmation of the optimized method were assessed by observing the retention time and spectrum of the peak of gossypol standard (40μg/mL) (Fig 5A), cottonseed sample spiked with standard gossypol (Fig 5B), and cottonseed extract (Fig 5C). Visual data prove that the method is specific to analyse free gossypol in cottonseed samples.

Linearity was evaluated by injection of eight concentrations of gossypol standard, in the range of 0.5 μg/mL to 100 μg/mL. Regression lines were constructed, and plots were visually and statistically evaluated. The proposed RP-UPLC-PDA method showed good linearity with R^2^>0.999. Residuals were randomly distributed around the fit line, indicating data was well adjusted to the regression model.

LoD is defined as lowest amount of analyte in a sample which can be detected, with LoQ the lowest amount of analyte in a sample which can be quantitatively determined with suitable precision and accuracy [65]. A limit of detection was observed at 0.2 μg/mL, with a limit of quantitation at 0.5 μg/mL.

The precision validation parameter employed was the closeness of agreement between a series of measurements obtained from multiple sampling of the same homogeneous sample under the prescribed conditions [65]. The method was repeatable for determination of free gossypol in cottonseed samples, with RSD <10% for each of replicate determination series. ANOVA-based analysis indicated that gossypol amounts in samples were found to be statistically equivalent in the three determination series.

Accuracy of a measurement result refers to the closeness of agreement between the measured value and the true value [66]. The spike recovery method is one of the most common technique for determining accuracy parameters during analysis of natural products. The accuracy of the UHPLC method was determined by a recovery study. The percent recovery determined fell within the range of 94 to 112%. Recovery values of 109%, 112% and 94% were observed for low, medium and high concentrations, respectively.

### Sample analysis

**Fig 6:**
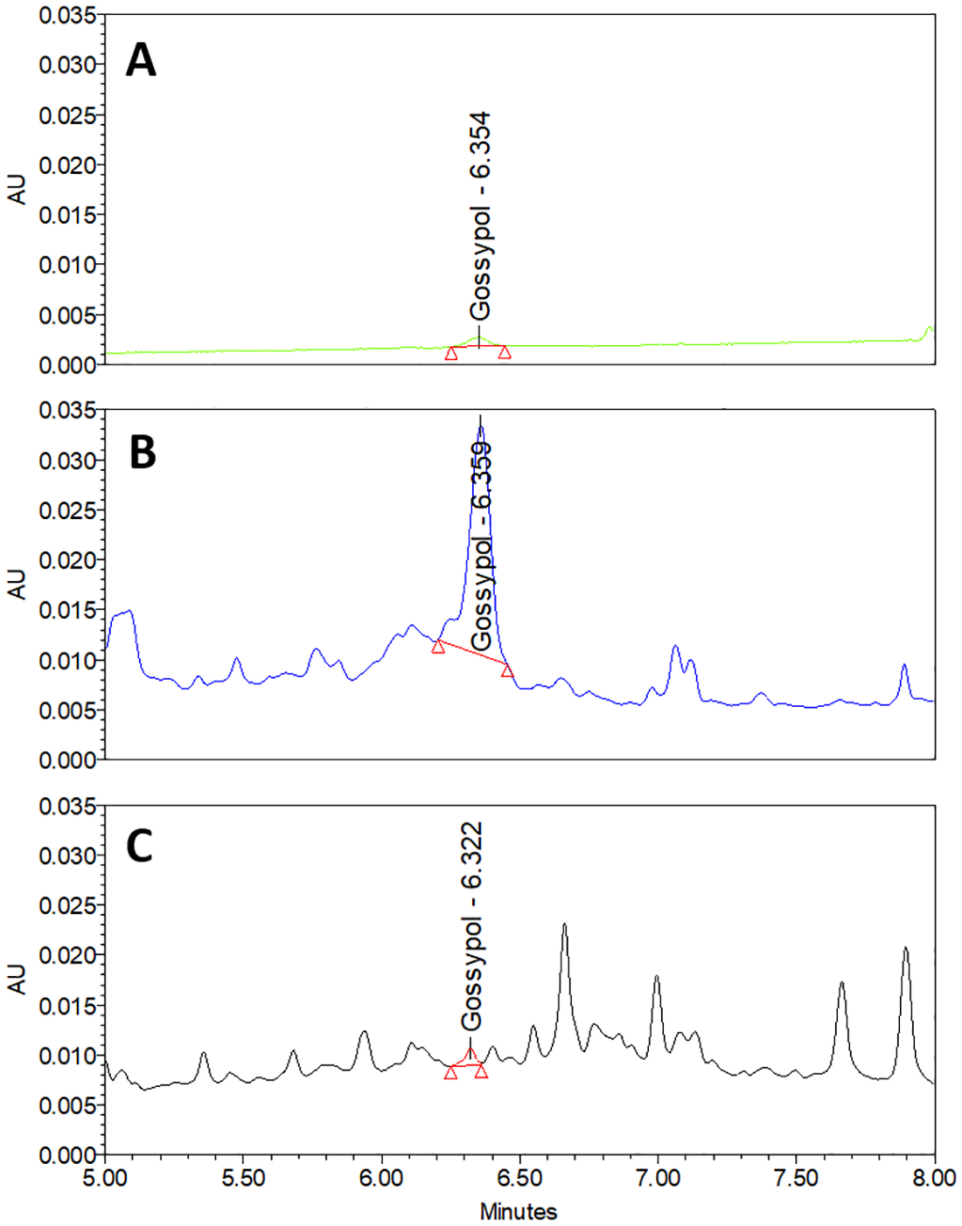
UHPLC chromatogram for standard gossypol and free gossypol (FG) extracts from CSC and FCSC. (A) Standard gossypol (0.5 μg/mL) (peak area = 4365); (B) FG from CSC extraction. (peak area = 141266); (C) FG from FCSC extraction (peak area = 4830). Gradient elution conditions were equal for all samples, with extraction conditions identical for CSC and FCSC samples.

The strain of the *Pleurotus* sp. fully colonized the autoclaved CS and CSC samples over the 30-day incubation period, confirming its ability to grow on cottonseed substrates in accord with previous observations [67,68]. The optimized extraction and quantification method developed for FG was able to efficiently detect residual traces of FG in both FCS and FCSC. Although additional peaks appeared following fungal fermentation of CSC, which could potentially interfere in the detection and quantification of FG, the method employed for integration of peaks was able to identify gossypol at the correct retention time, without obstruction by any other peak (Fig 6). This identification was also confirmed on the basis of the UV spectrum of the peak. No deviation of the retention time was noted for gossypol when eluted from different sample materials, with a constant period observed from standard gossypol (Fig 6A), non-fermented samples (Fig 6B) and fermented samples (Fig 6C).

The significant reduction in FG in the autoclaved substrates subjected to fermentation is indicative of the efficiency of the biological detoxification of gossypol by macrofungal species such as *Pleurotus*. The percentage of free gossypol remaining in these fermented sample, when compared to values in the original (non-treated) samples were 0.6% and 6.1% for FCS and FCSC, respectively. In contrast, cottonseed samples subjected only to autoclaving, maintained relatively high FG values, at 48% for CS and 23.75% for CSC. Based on the results summarized in Table 2, the method for FG extraction and quantification was appropriate for detection of FG not only in non-treated and autoclaved CS and CSC, but also in the autoclaved then fermented substrates FCS and FCSC, revealing the suitability of the method for FG detection at trace levels.

**Table 2:** Free gossypol concentrations detected in different samples.

Samples	Free gossypol (mg/kg)
**CS**	4886.84 ± 6.85
**Autoclaved CS**	2345.50±14.76
**FCS**	15.26 ± 2.23
**CSC**	56.88 ± 2.98
**Autoclaved CSC**	13.51±0.57
**FCSC**	0.847± 0.078

CS (cottonseed); FCS (Fermented Cottonseed); CSC (Cottonseed cake); FCSC (Fermented Cottonseed cake).

## Discussion

The typically high concentrations of FG observed in CSC after cotton oil extraction are a limiting factor in the application of cotton biomass as feed for livestock [11, 69]. This applies not only to non-ruminants such as pigs, birds, fish, and rodents, but also to ruminants such as sheep and goats [7,70]. As each animal species will vary in its’ tolerance to FG [69], and as concentrations in cottonseed cake differ according to cotton plant variety and oil extraction method employed [7,53], efficient detoxification methods are required to guarantee elimination of the toxic compound for safe application of CSC as feed for different livestock species. In this context, accurate and sensitive quantitation methods for FG in such biomass materials are essential [71].

Whilst chemical and physical approaches can be employed for detoxification of CSC, there are disadvantages in such methods, with further processing required to eliminate chemical residues and loss of biomass protein content common [57,72]. Such problems can be avoided, however, through biological treatments for CSC detoxification. Considerable promise lies in the application of filamentous fungi, with basidiomycete white rot fungi effectively employed in CSC detoxification [31,33,34]. Given the need for a highly sensitive, rapid and specific technique to analyze FG in FCSC, an optimized UHPLC method was developed in this study, suitable for efficient and rapid extraction and trace level quantification of FG in cottonseed cake following detoxification by fungi.

The method developed here for FG guarantees the preservation of this unstable molecule throughout the extraction and UHPLC detection procedure. In comparison with previously published liquid chromatographic methods for detection of gossypol, as summarized in Table 3, this RP-UHPLC-PDA method provides a lower limit of detection and quantification for analysis of cotton samples. The chromatogram generated by UHPLC, which requires a shorter run time and offers enhanced sensitivity [73], showed very narrow well-defined peaks, allowing quantification and detection limits of 0.5 and 0.2 μg/mL, respectively. When compared to Cai et al. [41] for example, who reported a minimum detection limit of 3 μg/mL for gossypol, the present method demonstrated an improved performance for free gossypol analysis.

**Table 3:** Comparison of different liquid chromatographic methods for analysis of free gossypol.

Cotton material	Extraction solvent	Equipment/ Column	Elution time(min)	Detector/ absorbance(nm)	Injection volume (μL)	LoD/LoQ*(linear range)	Reference
Seed, Cottonseed cake and Fungi fermented cottonseed meal	70% acetone	UHPLC/ KINETEX^®^ C18 (100 x 2.1 mm, 2.6 μm	14	PDA/254	2	0.4/1ng (1-200ng)	Present method
Seed, leaves, and flower buds	Ethanol-water-ether-acetic acid (59:24:17:0.2)	HPLC/ Scientific Glass Engineering LC-18 (4.6 mm X 25 cm, 5 μm)	NA	PDA/272	20	NA/25ng(25-6000ng)	[74]
Seed, stems and roots	95% ethanol-deionized water-diethyl ether-glacial acetic acid	HPLC/ C18 Cartridge	NA	UV/254	50	NA/5ng (5-2000ng)	[58]
Seed	95% ethanol-distilled water-diethyl ether-glacial acetic acid.	HPLC/μBondapak C18 (30 cm x 3.9 mm)	30	UV/254	25	NA/10ng (10-10000ng	[39]
Cotton materials (seed)	Acetone	HPLC/ Hewlett-Packard Zorbax Eclipse XDB-C18 (4⋅6 mm × 250 mm, 5 μm)	13.5	UV/254	5	NA/15ng (15-300ng)	[42]
Seeds	70% Acetone	HPLC/ Waters NOVA- PAK C18 (3.9 mm X 15 cm, 4 μm)	10	UV/254	50	NA/50ng (50-5000ng)	[60]
Seeds	80% acetonitrile-0.1% phosphoric acid	HPLC/Waters Novapak C18 (150 mm x 3.9 mm, 4 μm)	30	UV/272 and 254	20	140ng/NA(NA)	[75]
Stem, root, seed or seed cake	Acetone	HPLC/ Lichrosorb-C18 (250 x 4.0 mm, 10 μm)	NA	UV/254	4	NA/100ng (100-800ng)	[41]

NA: data not available;

*original values were transformed to ng after calculation based on injection volume.

Previous studies have reported that the stability of gossypol can be affected by the extraction solvent employed [40,41]. Here, 70% acetone was used successfully for efficient extraction. At this concentration, solubility of free gossypol is known to be high [41], with the compound also remaining stable [58]. When testing 100% acetone to extract spiked gossypol on CSC (Fig 1B), by contrast, the presence of an extra peak on the chromatogram indicated the likely conversion of gossypol to another organic group. Such an extra peak has also been reported in experiments related to photodecomposition [76]. This extra peak is likely to be similar in structure to that of the gossypol molecule, since a correlated spectrum can be observed (Fig 2). Previous attempts employing acetonitrile as solvent resulted in less efficient extraction of FG, with this compound unstable in several solvents, including acetonitrile, when maintained at 37°C or at room temperature [58]. As gossypol has been widely reported to be unstable in alcohols [38, 39, 56], this class of solvent was not tested in the current study for FG extraction.

Elevated temperatures are also known to cause degradation of gossypol [27,58]. In order to prevent such degradation during extraction, sonication was performed on ice, with low temperatures employed during centrifugation steps, room temperature conditions used for evaporation via speed vacuum, and low temperatures (8°C) employed for sample maintenance during UHPLC.

A two-step extraction procedure using a final concentration of 70% aqueous acetone (following application of 3 mL water, followed by 7 mL acetone) was demonstrated to be more appropriate for extraction than a one step process (70% aqueous acetone solution). Apparently, sonication in water facilitates rupture of the pigment glands in cotton, allowing FG to be released before solubilisation in acetone [77].

Centrifugation of the sample allowed a separation of the FG dissolved in the acetone solution from the solid CSC mass, in contrast to published assays that employ filtration with filter paper at this step [38,43,78,79]. Centrifugation is more appropriate, as evaporation of the solvent, or sample oxidation are then avoided. Similarly, sample concentration in a speed vacuum at room temperature caused less degradation of FG than when compared to rotary evaporation at 40°C.

Attempts at extraction using different columns, mobile phases and isocratic elution resulted in a non-satisfactory broad peak. By contrast, the KINETEX^®^ RP-C18 column gradient elution with methanol:water (TFA 0.1%) resulted in a sharper peak, with high sensitivity for gossypol detection. Although alcoholic solvents are known to increase the instability of gossypol [58], elution in alcoholic systems using short retention times does not negatively affect accurate gossypol detection [40,41]. Under such conditions, with gradient elution, methanol was successfully employed as mobile phase in this method. The typically observed formation of a broad peak during gossypol elution may be due to the partial ionization of the molecule, which can be reduced by adding 0.1% of phosphoric acid in the elution system [39]. In this study, 0.1% TFA, which shows the same efficiency of phosphoric acid, was employed to reduce such ionization. The short elution time required when using the KINETEX^®^ column also supports a rapid and economically efficient analysis of large numbers of samples.

## Conclusion

The optimized extraction procedure developed was able to efficiently extract FG from cottonseed and its derivatives, maintaining molecule stability. The RP-UHPLC-PDA detection method developed enabled rapid (14 minutes) and sensitive (quantitation limits: 0.2 μg/mL) determination and quantification of low concentration FG from FCSC. This procedure will facilitate accurate analysis of the efficiency of methods for biodetoxification of FG to trace levels using promising microorganisms such as basidiomycete fungi.
